# Supplementary material for: Multi-domain and complex protein structure prediction using inter-domain interactions from deep learning
Source: Commun Biol. 2023 Dec 1;6:1221. doi: 10.1038/s42003-023-05610-7 (PMC10692239; doi:10.1038/s42003-023-05610-7)
Supplement: Supplementary file 1 — Supplementary Information [file 42003_2023_5610_MOESM1_ESM.pdf]

Supplementary Information for

Multi-domain and complex protein structure prediction using inter-domain interactions from deep learning

Yuhao Xia[1,2], Kailong Zhao[1,2], Dong Liu[1], Xiaogen Zhou[1], and Guijun Zhang[1,*]

[1] College of Information Engineering, Zhejiang University of Technology, HangZhou 310023, China.

[2] These authors contributed equally: Yuhao Xia, Kailong Zhao.

[*] Correspondence should be addressed to Guijun Zhang (zgj@zjut.edu.cn).

Supplementary Notes

Supplementary Note 1. Description of evaluation metrics

RMSD. RMSD ($=\sqrt{\frac{1}{N}\sum_{i=1}^{N} d_i^2}$) is calculated as an average of distance error (d_i) with equal weight over all residue pairs. The lower value indicates closer structural similarity.

TM-score. TM-score[1] is a metric for evaluating the topological similarity between protein structures, which can be calculated by

$$\text{TM-score} = \max\left[\frac{1}{N_{\text{res}}}\sum_{i=1}^{N_{\text{aligned}}}\frac{1}{1+\left(\frac{d_i}{d_0(N_{\text{res}})}\right)^2}\right] \qquad (S1)$$

where N_{res} is the amino acid sequence length of the target protein, N_{aligned} is the length of the aligned residues to the reference (native) structure, d_i is the distance between the i-th pair of aligned residues, $d_0(N_{\text{res}}) = 1.24\sqrt[3]{N_{\text{res}}-15}-1.8$ is a scale to normalize the match difference, and 'max' refers to the optimized value selected from various rotation and translation matrices for structure superposition. The value of TM-score ranges in (0,1], where a higher value indicates closer structural similarity. Stringent statistics showed that TM-score >0.5 corresponds to a similarity with two structures having the same fold and/or domain orientations[2].

DockQ. DockQ[3] is a score in the range [0,1] that can be used to measure the quality of the interface. Interface with score greater than 0.23 is considered as successfully predicting the interface, greater than 0.49 and less than 0.8 is considered as medium quality, and greater than 0.8 is considered as high quality. DockQ score is calculated as:

$$DockQ(F_{\text{nat}}, LRMS, iRMS, d_1, d_2) = (F_{\text{nat}} + RMS_{\text{scaled}}(LRMS, d_1) + RMS_{\text{scaled}}(iRMS, d_2))/3 \qquad (S2)$$

where F_{nat} is the fraction of native interfacial contacts preserved in the interface of the predicted complex. LRMS is the Ligand Root Mean Square deviation calculated for the backbone of the shorter chain (ligand) of the model after superposition of the longer chain (receptor)[4]. iRMS, the receptor-ligand interface in the target (native) is redefined at a relatively relaxed atomic contact cutoff of 10Å which is twice the value used to define inter-residue 'interface' contacts in case of F_{nat}. The backbone atoms of these 'interface' residues are then superposed on their equivalents in the predicted complex (model) to compute the iRMS[4]. $RMS_{\text{scaled}}(RMS, d_i)$ is defined as:

$$RMS_{\text{scaled}}(RMS, d_i) = \frac{1}{1+\left(\frac{RMS}{d_i}\right)^2} \qquad (S3)$$

which represents the scaled RMS deviations corresponding to any of the two terms, LRMS or iRMS (RMS) and d_i is a scaling factor, d_1 for LRMS and d_2 for iRMS, optimized to d_1 = 8.5Å and d_2 = 1.5Å.

Inter-domain distance error. We define the inter-domain distance error, err_{dist}, to evaluate the predicted inter-domain distance precision, which is calculated as the errors (Å) between the predicted inter-domain distance and the true inter-domain distance extracted from experimental structure, with smaller value indicating higher predicted distance precision.

$$err_{\text{dist}} = \frac{1}{N_{\text{pair}}}\sum_{(i,j)}\left|d_{(i,j)}^{\text{pre}} - d_{(i,j)}^{\text{true}}\right|, \ \forall(i,j) \in \mathcal{S}_{\text{inter domain}}, \ i<j \qquad (S4)$$

where $d_{(i,j)}^{\text{pre}}$ and $d_{(i,j)}^{\text{true}}$ are the predicted distance and the true distance of inter-domain residue pair (i,j), respectively. $\mathcal{S}_{\text{inter domain}}$ represents the set of inter-domain residue pairs, and N_{pair} is the number of inter-domain residue pairs.

Supplementary Note 2. Establishment of residual local coordinate system

We establish the residual local coordinate system using three atoms Cα, C and N in the protein structure through a Gram-Schmidt process. We take Cα as the center of the local coordinate system, and refer to the positions of N, Cα and C as $\vec{\mathbf{x}}_1$, $\vec{\mathbf{x}}_2$ and $\vec{\mathbf{x}}_3$.

$$\vec{\mathbf{v}}_1 = \vec{\mathbf{x}}_3 - \vec{\mathbf{x}}_2 \tag{S5}$$

$$\vec{\mathbf{e}}_1 = \vec{\mathbf{v}}_1 / \|\vec{\mathbf{v}}_1\| \tag{S6}$$

$$\vec{\mathbf{v}}_2 = \vec{\mathbf{x}}_1 - \vec{\mathbf{x}}_2 \tag{S7}$$

$$\vec{\mathbf{v}}_3 = \vec{\mathbf{e}}_1 \times \vec{\mathbf{v}}_2 \tag{S8}$$

$$\vec{\mathbf{e}}_3 = \vec{\mathbf{v}}_3 / \|\vec{\mathbf{v}}_3\| \tag{S9}$$

$$\vec{\mathbf{e}}_2 = \vec{\mathbf{e}}_3 \times \vec{\mathbf{e}}_1 \tag{S10}$$

$$\mathbf{R} = [\vec{\mathbf{e}}_1, \vec{\mathbf{e}}_2, \vec{\mathbf{e}}_3] \tag{S11}$$

$$\vec{\mathbf{t}} = \vec{\mathbf{x}}_2 \tag{S12}$$

$$\mathbf{A} = \begin{bmatrix} \mathbf{R} & \vec{\mathbf{t}} \\ \mathbf{0} & 1 \end{bmatrix} \tag{S13}$$

where $\mathbf{A}$ is the affine transformation from the residual local coordinate system to the ground coordinate system, $\mathbf{R}$ and $\vec{\mathbf{t}}$ are the rotation matrix and the translation vector, respectively.

Supplementary Note 3. Equivalent conversion of rotation and translation

We calculate the rotation matrix and the translation vector between two residual local coordinate system spaces as follows:

$$\mathbf{R}_{(i,j)} = \mathbf{R}_i^{-1}\mathbf{R}_j \tag{S14}$$

$$\vec{\mathbf{t}}_{(i,j)} = \mathbf{R}_i^{-1}(\vec{\mathbf{t}}_j - \vec{\mathbf{t}}_i) \tag{S15}$$

The three Euler angles (α, β, γ) are calculated as:

$$\alpha = \arctan\left({}^{r_{32}}\!/_{r_{33}}\right) \tag{S16}$$

$$\beta = \arctan\left({}^{-r_{31}}\!\Big/_{\sqrt{r_{32}^2 + r_{33}^2}}\right) \tag{S17}$$

$$\gamma = \arctan\left({}^{r_{21}}\!/_{r_{11}}\right) \tag{S18}$$

The distance r, polar angle θ and azimuthal angle ϕ are calculated as:

$$r = \sqrt{t_1^2 + t_2^2 + t_3^2} \tag{S19}$$

$$\theta = \arccos\left({}^{t_3}\!\Big/_{\sqrt{t_1^2 + t_2^2 + t_3^2}}\right) \tag{S20}$$

$$\phi = \arctan\left({}^{t_2}\!/_{t_1}\right) \tag{S21}$$

Supplementary Note 4. The influence of the number of movable residues near the domain boundaries on the final structure

In order to investigate the influence of the number of movable residues near the domain boundaries on the final structure, we assemble the full-length structure using 4 and 8 residues near the domain boundaries, respectively. The result shows that the average TM-score is improved from 0.916 to 0.922 by using 8 residues near the domain boundaries (see table). In addition, the number of models with TM-score >0.9 also increased from 173 to 178. We show a case of three-domain protein (PDB ID: 1GRI_A) which is made up of three domains with two long loops. It can be seen from the figure that the DeepAssembly model by using 8 movable residues in the linker is more accurate than using 4 residues (the TM-score improved from 0.752 to 0.958). The linker in the predicted structure is broken and there is a large deviation compared with the experimental structure (shown in the yellow boxes). However, by increasing the number of movable residues in linker, the inter-domain orientation of the predicted structure is not only improved, but the linker's shape is also closer to the experimental structure. This suggests that more movable residues near the domain boundaries may increase the flexibility of the linker during optimization, making it easier to sample the correct inter-domain orientation, thereby improving the prediction accuracy. Therefore, we set the number of movable residues near the domain boundaries to 8 in the domain assembly module of DeepAssembly.

Table. The performance of DeepAssembly for assembling the full-length structure using 4 and 8 residues near the domain boundaries, respectively. #TM-score>0.9 represents the number of models with TM-score > 0.9.

Method	RMSD (Å)	TM-score	#TM-score>0.9
DeepAssembly (4 residues)	3.12	0.916	173
DeepAssembly (8 residues)	2.91	0.922	178

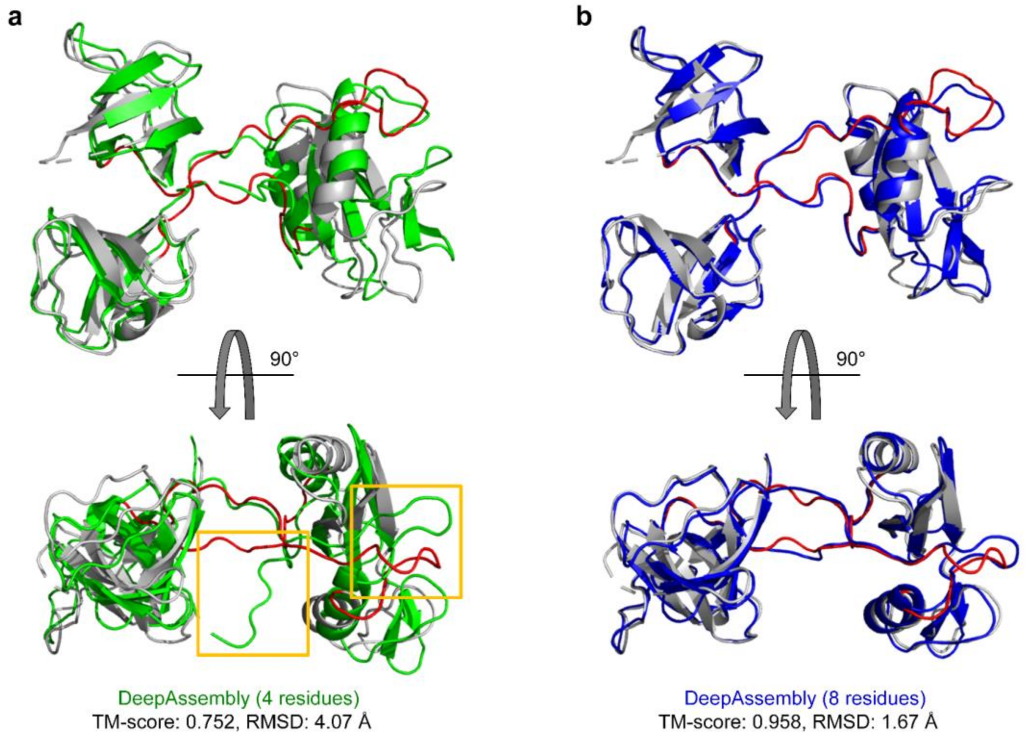

Figure. The case of a three-domain protein (PDB ID: 1GRI_A) which is made up of three domains with two long loops. Experimental structure is colored in gray, and the predicted models of DeepAssembly using 4 and 8 residues near the domain boundaries are colored in green (a) and blue (b), respectively. The two long loops connecting the three domains in the experimental structure are colored in red.

Supplementary Tables

Supplementary Table 1. Summary of the performance of DeepAssembly and AlphaFold2 for predicting multi-domain proteins. #TM-score>0.9 represents the number of models with TM-score > 0.9.

Method	RMSD (Å)	TM-score	#TM-score>0.9
DeepAssembly	2.91	0.922	178
DeepAssembly (AF2 domain)	3.11	0.919	176
AlphaFold2	3.58	0.900	166

Supplementary Table 2. Results of AlphaFold2 and DeepAssembly on CASP14 and CASP15 targets.

Method	CASP14		CASP15	
	RMSD (Å)	TM-score	RMSD (Å)	TM-score
AlphaFold2	7.45	0.832	20.58	0.567
DeepAssembly	6.68	0.850	15.94	0.584

Supplementary Table 3. Results of DeepAssembly, AlphaFold-linker and RoseTTAFold on the heterodimers. Acceptable: 0.23 ≤ DockQ < 0.49, Medium: 0.49 ≤ DockQ < 0.80, High: DockQ ≥ 0.80.

Method	Success rate (SR) (%)	Acceptable Count	Medium Count	High Count
DeepAssembly	32.4	21	48	11
AlphaFold-linker	40.9	31	51	19
RoseTTAFold	18.6	28	17	1

Supplementary Table 4. TM-score of the models assembled by DeepAssembly, SADA, DEMO and AIDA using experimental domain structures. “2dom”, “3dom”, and “m4dom” represent the classification of proteins with two, three, and more than four domains, respectively.

Method	2dom		3dom		m4dom		all	
	average	median	average	median	average	median	average	median
DeepAssembly	0.896	0.985	0.851	0.984	0.725	0.840	0.856	0.976
SADA	0.840	0.903	0.709	0.688	0.582	0.555	0.763	0.782
DEMO	0.779	0.782	0.649	0.628	0.517	0.504	0.702	0.698
AIDA	0.671	0.662	0.514	0.497	0.424	0.405	0.589	0.581

Supplementary Table 5. Summary of errors (Å) in inter-domain distances predicted by different methods. "2dom", "3dom", and "m4dom" represent the classification of proteins with two, three, and more than four domains, respectively.

Method	2dom		3dom		m4dom		all	
	average	median	average	median	average	median	average	median
DeepAssembly	0.629	0.344	0.515	0.396	0.383	0.251	0.560	0.343
AlphaFold2	0.833	0.462	0.597	0.520	0.548	0.483	0.724	0.476
RoseTTAFold	1.252	1.029	1.028	0.943	0.990	0.951	1.151	0.984

Supplementary Table 6. Ablation results of inter-domain distance prediction accuracy. "2dom", "3dom", and "m4dom" represent the classification of proteins with two, three, and more than four domains, respectively.

Model no.	Input features	2dom	3dom	m4dom	all
1	All	0.629	0.515	0.383	0.560
2	No inter-domain features	0.654	0.487	0.463	0.580
3	No PAthreader (use HHsearch templates)	0.692	0.571	0.658	0.655
4	No templates	1.700	1.492	1.551	1.621

Supplementary Table 7. Average errors of intra-/inter-domain distance and orientation predicted by AffineNet. α, β, γ, θ, ϕ are angles representing the inter-residue orientations.

	Distance error (Å)	Orientation error (rad)				
		α	β	γ	θ	ϕ
Inter-domain	0.560	0.394	0.255	0.352	0.206	0.261
Intra-domain	0.453	0.306	0.180	0.261	0.148	0.204

Supplementary Table 8. Input features to the model. Feature dimensions: N_{res} is the number of residues, and N_{templ} is the number of templates.

Type	Feature	Shape	Description
MSA features	Amino acid type	[N_{res}, 21]	One-hot representation of the input amino acid sequence (20 amino acids + unknown).
	Position-specific scoring matrix	[N_{res}, 21]	The matrix representing the amino acid frequency at specific position in the MSA (20 amino acids + gap).
	Positional entropy	[N_{res}, 1]	A measure representing the amino acid conservation.
	Inverse covariance matrix	[N_{res}, N_{res}, 441]	The matrix representing the co-evolutionary information between residues.
Template features	Template inter-residue rotation	[N_{templ}, N_{res}, N_{res}, 63]	One-hot pairwise feature indicating the three Euler angles (α, β, γ) converted by rotation matrix between local coordinate systems in residues. Euler angle α is discretized into 24 bins of equal width between -180° and 180°; β is discretized into 12 bins of equal width between -90° and 90°; γ is discretized into 24 bins of equal width between -180° and 180°; and one more bin in each angle represents no-contact with larger distance.
	Template inter-residue translation	[N_{templ}, N_{res}, N_{res}, 75]	One-hot pairwise feature indicating spherical coordinate system (r, θ, ϕ) converted by translation vector between local coordinate systems in residues. The pairwise distance r is discretized into 36 bins of equal width between 2 Å and 20 Å; polar angle θ is discretized into 12 bins of equal width between 0° and 180°; azimuthal angle ϕ is discretized into 24 bins of equal width between -180° and 180°; and one more bin in each represents any larger distance.
Inter-domain features	Inter-domain contact	[N_{res}, N_{res}, 1]	The feature indicating if there is a contact between a pair of residues.
	Inter-domain mask	[N_{res}, N_{res}, 1]	A mask indicating whether a pair of residues are in different domains.

Supplementary Figures

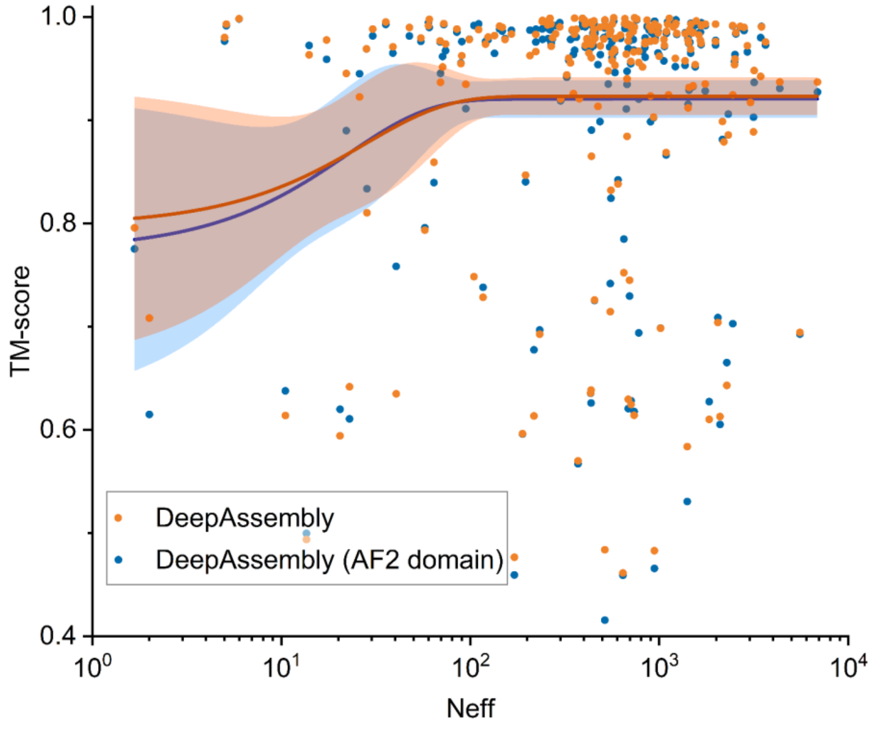

Supplementary Fig. 1 Scatterplot of the TM-score of models predicted by DeepAssembly and DeepAssembly (AF2 domain) versus the number of effective sequences (Neff) in MSAs. The orange and blue curves are obtained by fitting the orange and blue dots with ExpDec1 function in Origin. The shaded area is the 95% confidence interval.

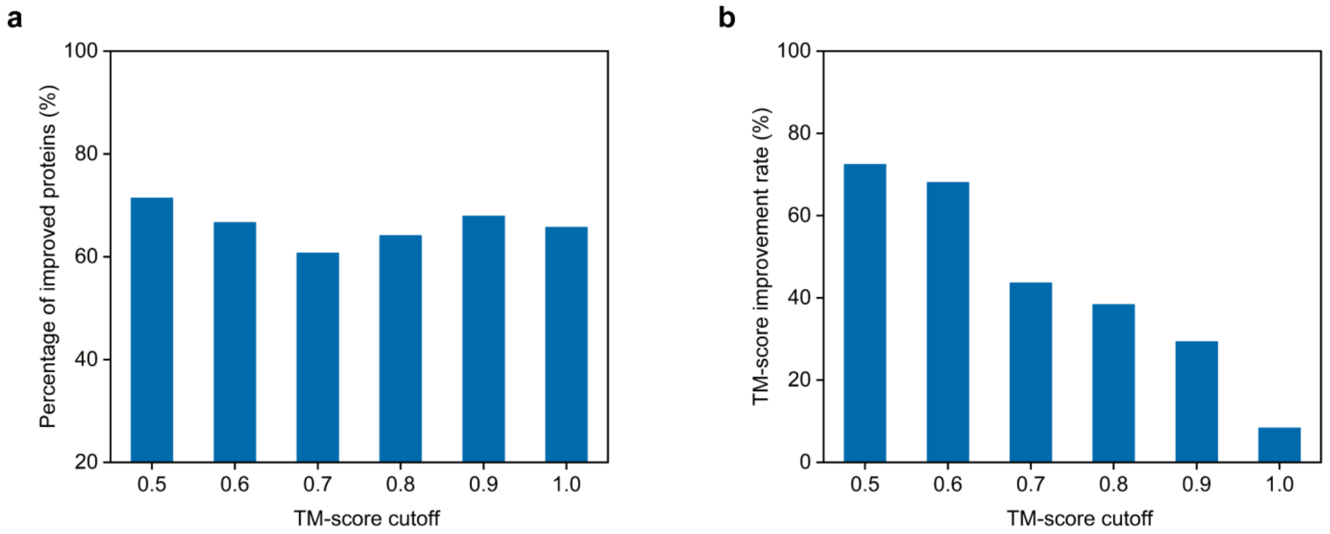

Supplementary Fig. 2 The improvement of DeepAssembly compared to AlphaFold2 prediction results. **a** The percentage of improvement by DeepAssembly on the corresponding proteins of the models predicted by AlphaFold2 with TM-score less than each cutoff. **b** The average TM-score improvement rate for the targets improved by DeepAssembly compared to AlphaFold2 at each TM-score cutoff.

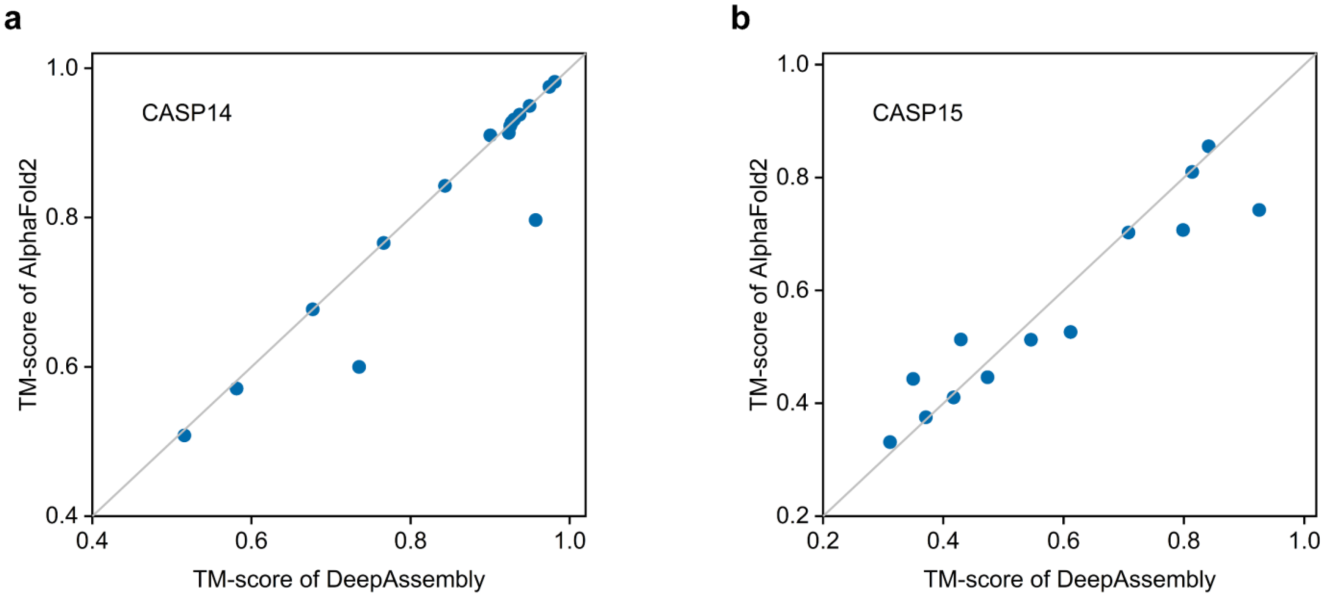

Supplementary Fig. 3 Head-to-head TM-score comparison of DeepAssembly with AlphaFold2. a Head-to-head comparison on each CASP14 target. **b** Head-to-head comparison on each CASP15 target.

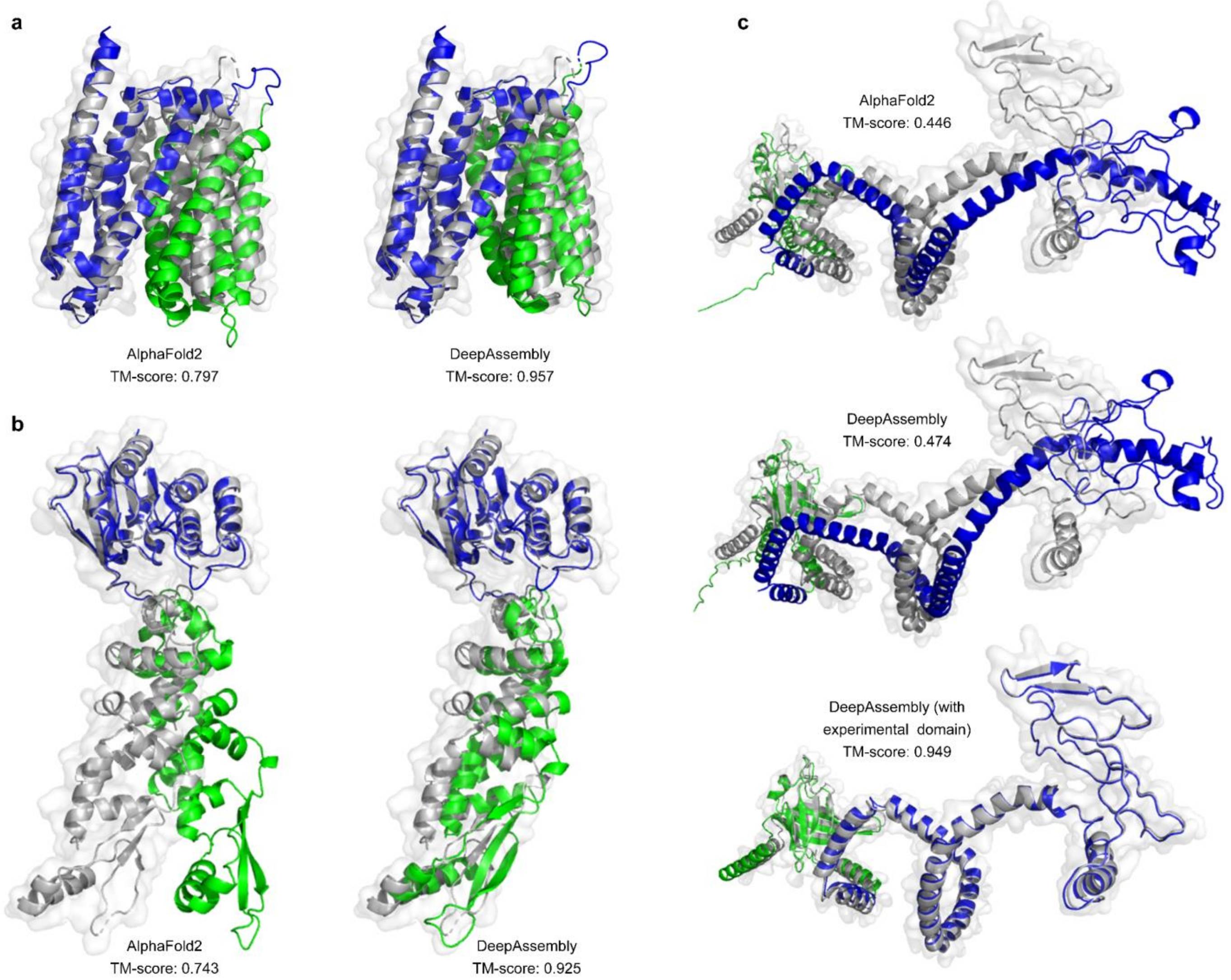

Supplementary Fig. 4 Examples of the CASP14 and 15 targets predicted by AlphaFold2 and DeepAssembly. The reference PDB structures are colored in gray, and the different domains of the predicted models are colored by blue and green. **a** T1024 (PDB ID: 6T1Z). **b** T1121 (PDB ID: 7TIL). **c** T1137s1 (PDB ID: 8FEF).

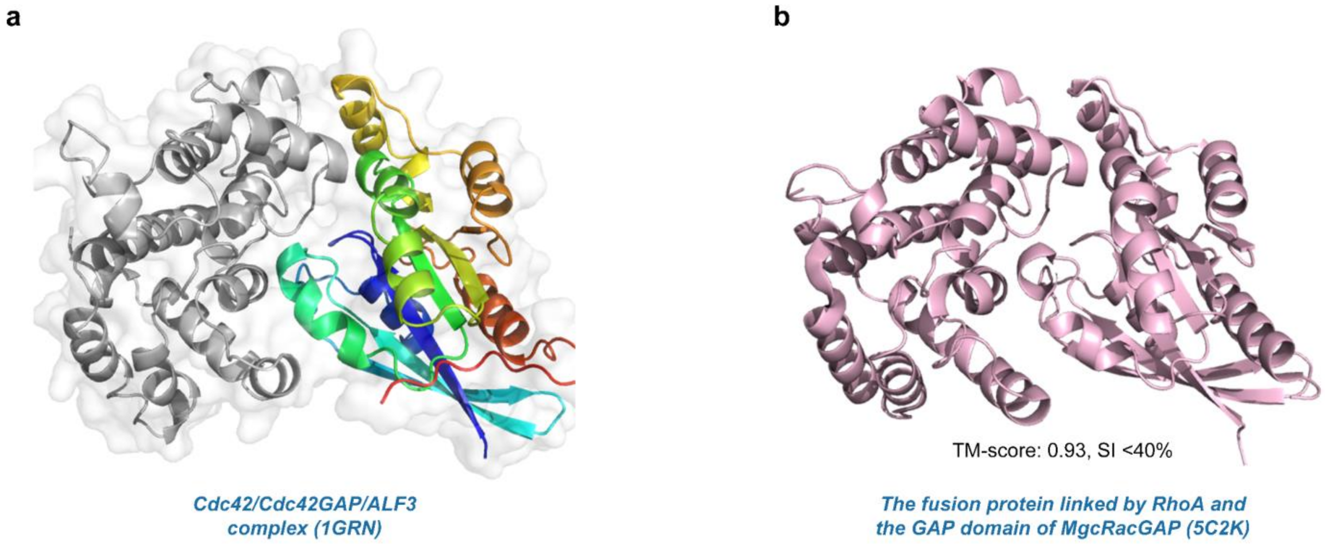

Supplementary Fig. 5 Cdc42/Cdc42GAP/ALF3 complex structure and its fusion protein template. a Crystal structure of the Cdc42/Cdc42GAP/ALF3 complex (PDB ID: 1GRN). Chain A is colored in a rainbow, and Chain B is colored in gray. **b** Crystal structure of the fusion protein linked by RhoA and the GAP domain of MgcRacGAP (PDB ID: 5C2K).

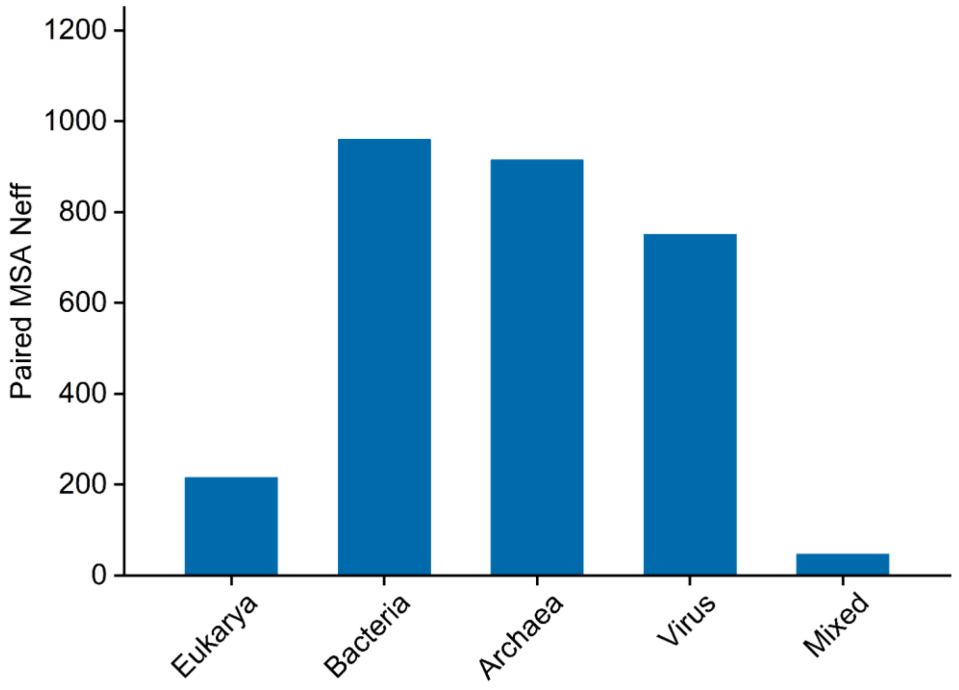

Supplementary Fig. 6 Paired MSA Neff for different kingdoms of the test set. The average Neff for each kingdom: Eukarya (215), Bacteria (959), Archaea (914), Virus (751), and mixed kingdoms (47).

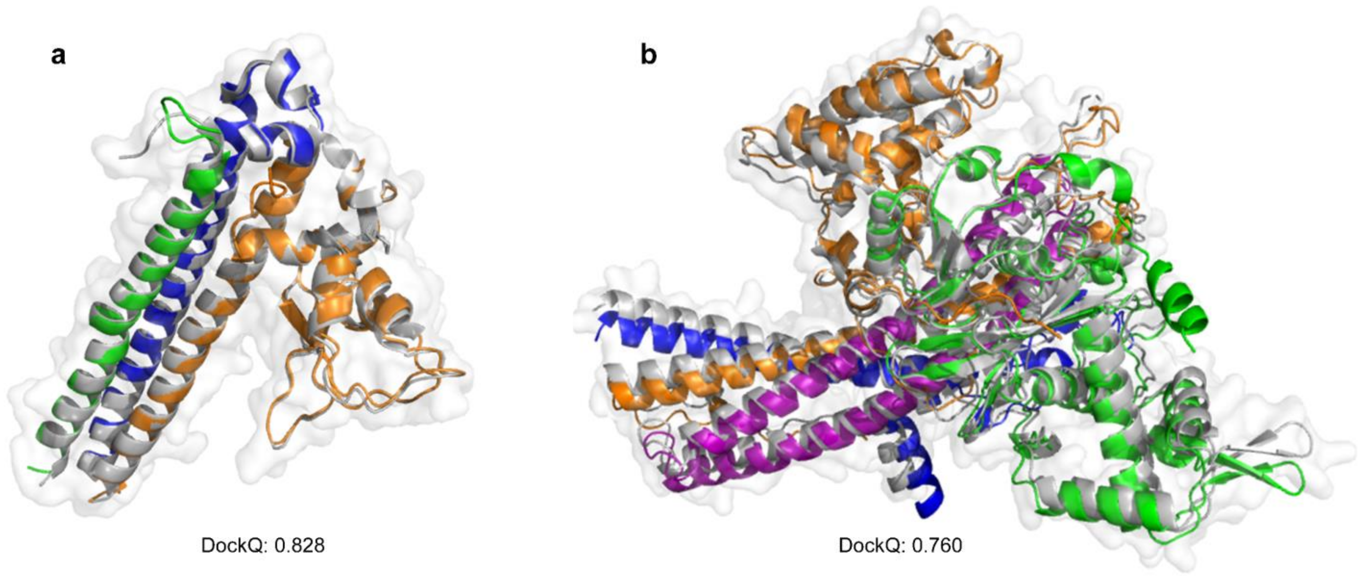

Supplementary Fig. 7 Structures generated by DeepAssembly for hetero-complexes with more than two chains. The reference PDB structures are colored in gray, and the different chains of the predicted models are colored by blue, green, orange, and purple. **a** Survivin-Borealin-INCENP core complex (PDB ID: 2QFA). **b** NuA4 core complex (PDB ID: 5J9T).

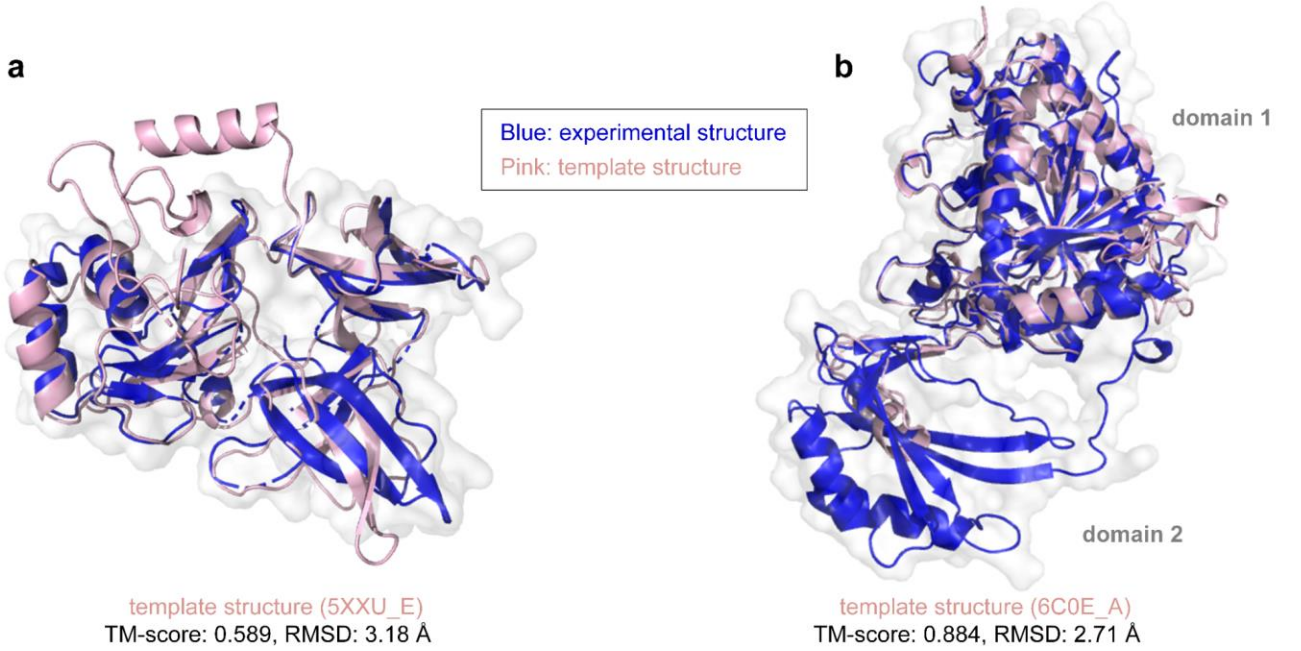

Supplementary Fig. 8 Template structures obtained by HHsearch for 5XXU_E and 6C0E_A. a For 5XXU_E, the best template structure searched by HHsearch has a TM-score of 0.589. **b** For 6C0E_A, the template for only one of its domains (domain 1) is searched by HHsearch, the other domain structure (domain 2) is missing from the template. Experimental structures are colored in blue, and the templates from HHsearch are colored in pink.

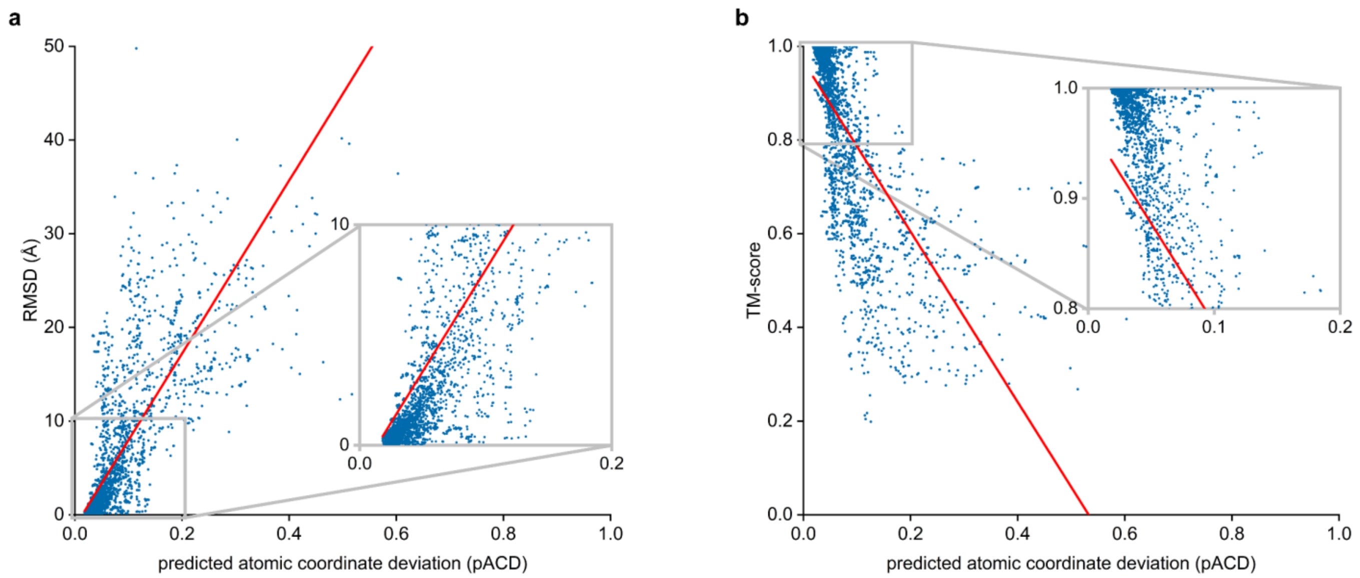

Supplementary Fig. 9 Correlation between predicted atomic coordinate deviation (pACD) and accuracy for 2,190 models predicted by DeepAssembly. a Correlation between pACD and full-chain RMSD (Pearson r=0.71, R^2=0.50). **b** Correlation between pACD and full-chain TM-score (Pearson r=-0.70, R^2=0.49).

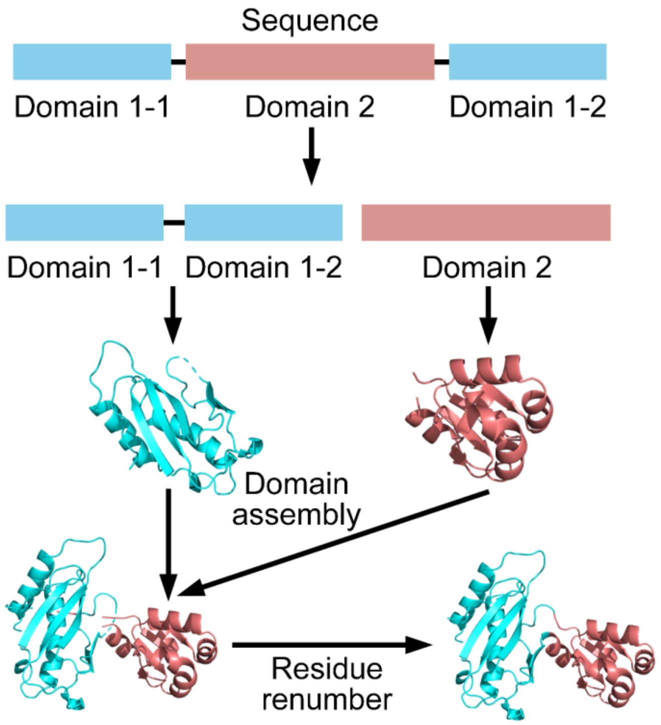

Supplementary Fig. 10 Diagram of the process for predicting the single-domain structure with disconnected sequences. Two single-domain structures are colored by blue and red, respectively.

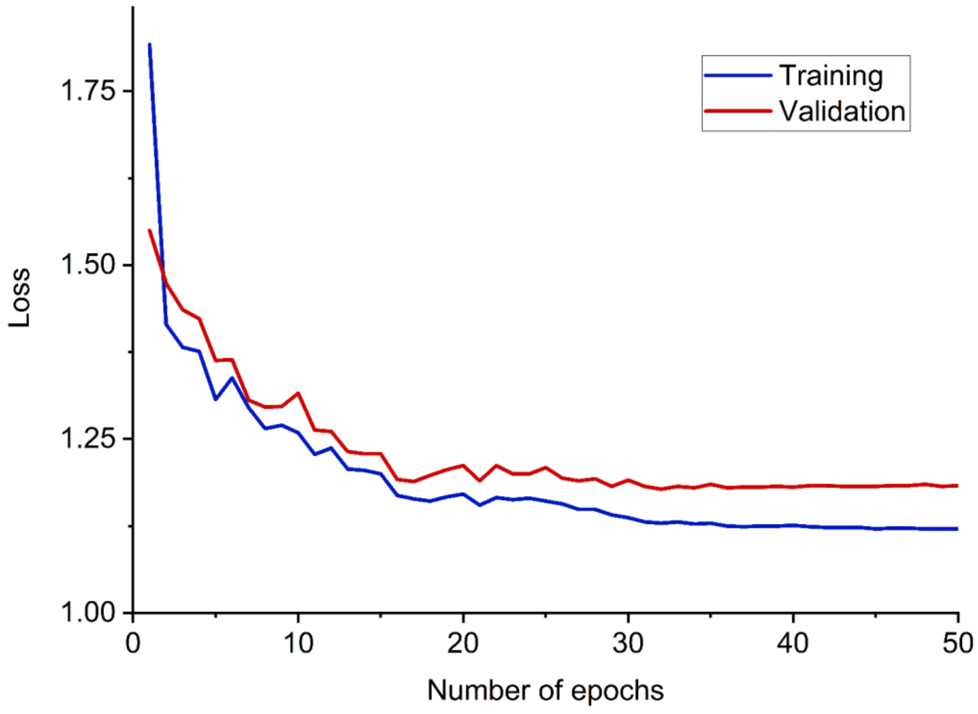

Supplementary Fig. 11 Learning curve of the network in training. The loss used here is the sum over the 6 individual cross-entropy losses with equal weight.

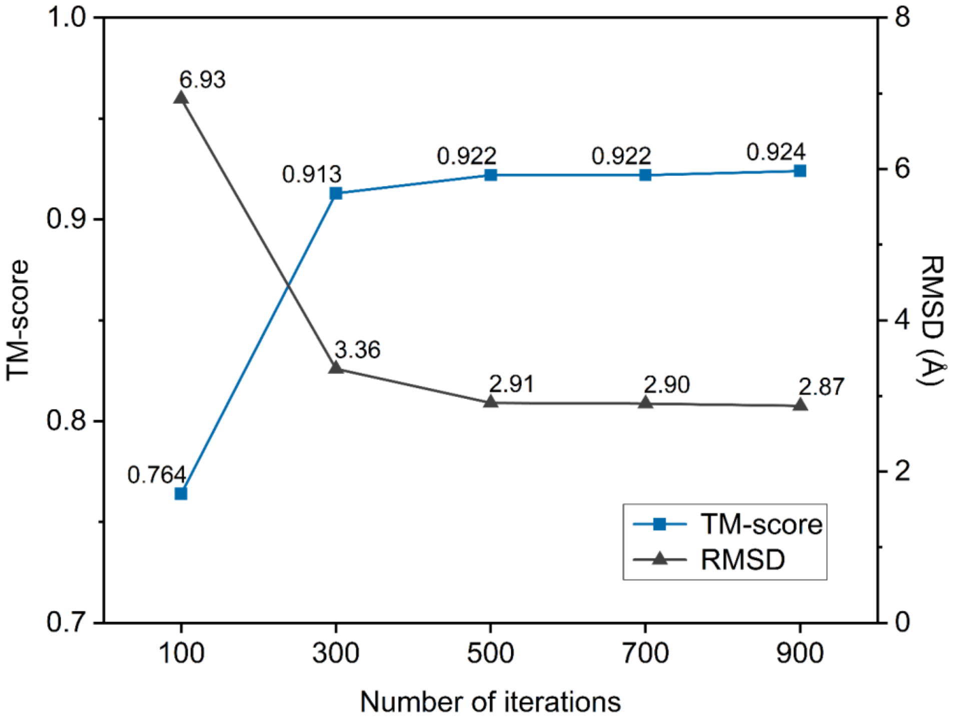

Supplementary Fig. 12 The average TM-score and RMSD of the final structures at different iteration numbers. The accuracy of the final structure increases as the number of iterations increases until it largely saturates when the number of iterations is greater than 500, at which point the optimization process gradually converges.

Supplementary References

1. Zhang, Y. & Skolnick, J. Scoring function for automated assessment of protein structure template quality. *Proteins* **57**, 702-710 (2004).
2. Xu, J. R. & Zhang, Y. How significant is a protein structure similarity with TM-score=0.5? *Bioinformatics* **26**, 889-895 (2010).
3. Basu, S. & Wallner, B. DockQ: A Quality Measure for Protein-Protein Docking Models. *PLoS ONE* **11**, e0161879 (2016).
4. Mendez, R., et al. Assessment of blind predictions of protein-protein interactions: Current status of docking methods. *Proteins* **52**, 51-67 (2003).
