# Supplementary material for: Multi-domain and complex protein structure prediction using inter-domain interactions from deep learning
Source: Commun Biol. 2023 Dec 1;6:1221. doi: 10.1038/s42003-023-05610-7 (PMC10692239; doi:10.1038/s42003-023-05610-7)
Supplement: Supplementary file 2 — Description of Additional Supplementary Files [file 42003_2023_5610_MOESM2_ESM.pdf]

Description of Additional Supplementary Files

File name: Supplementary Data 1
Description: The source data behind the Figures 2, 3, 8, 9 in the paper.

File name: Supplementary Data 2
Description: The source data behind the Figure 4 in the paper.

File name: Supplementary Data 3
Description: The source data behind the Supplementary Table 2 in the Supplementary Information.

File name: Supplementary Data 4
Description: The source data behind the Figures 5-6 in the paper.
