# Supplementary material for: Multi-domain and complex protein structure prediction using inter-domain interactions from deep learning
Source: Commun Biol. 2023 Dec 1;6:1221. doi: 10.1038/s42003-023-05610-7 (PMC10692239; doi:10.1038/s42003-023-05610-7)
Supplement: Supplementary file 7 — Reporting Summary [file 42003_2023_5610_MOESM7_ESM.pdf]

Corresponding author(s): Guijun Zhang
Last updated by author(s): Oct 15, 2023

Reporting Summary

Nature Portfolio wishes to improve the reproducibility of the work that we publish. This form provides structure for consistency and transparency in reporting. For further information on Nature Portfolio policies, see our Editorial Policies and the Editorial Policy Checklist.

Statistics

For all statistical analyses, confirm that the following items are present in the figure legend, table legend, main text, or Methods section.

n/a	Confirmed	
☐	☒	The exact sample size (*n*) for each experimental group/condition, given as a discrete number and unit of measurement
☐	☒	A statement on whether measurements were taken from distinct samples or whether the same sample was measured repeatedly
☐	☒	The statistical test(s) used AND whether they are one- or two-sided<br>*Only common tests should be described solely by name; describe more complex techniques in the Methods section.*
☒	☐	A description of all covariates tested
☐	☒	A description of any assumptions or corrections, such as tests of normality and adjustment for multiple comparisons
☐	☒	A full description of the statistical parameters including central tendency (e.g. means) or other basic estimates (e.g. regression coefficient) AND variation (e.g. standard deviation) or associated estimates of uncertainty (e.g. confidence intervals)
☐	☒	For null hypothesis testing, the test statistic (e.g. *F*, *t*, *r*) with confidence intervals, effect sizes, degrees of freedom and *P* value noted<br>*Give P values as exact values whenever suitable.*
☒	☐	For Bayesian analysis, information on the choice of priors and Markov chain Monte Carlo settings
☐	☒	For hierarchical and complex designs, identification of the appropriate level for tests and full reporting of outcomes
☐	☒	Estimates of effect sizes (e.g. Cohen's *d*, Pearson's *r*), indicating how they were calculated

Our web collection on statistics for biologists contains articles on many of the points above.

Software and code

Policy information about availability of computer code

Data collection	The online server of DeepAssembly is made freely available at http://zhanglab-bioinf.com/DeepAssembly/.
Data analysis	Data analysis used Python v3.6 (https://www.python.org/). TM-score v20220227 (https://zhanggroup.org/TM-score/) was used for computing RMSDs and TM-scores. DockQ v1.0 (https://github.com/bjornwallner/DockQ/) was used for computing DockQ scores. Structure visualizations were created in Pymol v2.4.0 (https://www.pymol.org).

For manuscripts utilizing custom algorithms or software that are central to the research but not yet described in published literature, software must be made available to editors and reviewers. We strongly encourage code deposition in a community repository (e.g. GitHub). See the Nature Portfolio guidelines for submitting code & software for further information.

Data

Policy information about availability of data

All manuscripts must include a data availability statement. This statement should provide the following information, where applicable:
- Accession codes, unique identifiers, or web links for publicly available datasets
- A description of any restrictions on data availability
- For clinical datasets or third party data, please ensure that the statement adheres to our policy

The authors declare that the data supporting the findings and conclusions of this study are available within the paper and its Supplementary Information file. Other data are available from the corresponding author upon reasonable requests.

Research involving human participants, their data, or biological material

Policy information about studies with [human participants or human data](). See also policy information about [sex, gender (identity/presentation), and sexual orientation]() and [race, ethnicity and racism]().

Reporting on sex and gender	Sex and gender were not considered in study
Reporting on race, ethnicity, or other socially relevant groupings	Race, ethnicity, or other socially relevant groupings were not considered in study
Population characteristics	There were no human study participants in the study
Recruitment	n/a
Ethics oversight	n/a

Note that full information on the approval of the study protocol must also be provided in the manuscript.

Field-specific reporting

Please select the one below that is the best fit for your research. If you are not sure, read the appropriate sections before making your selection.

☒ Life sciences ☐ Behavioural & social sciences ☐ Ecological, evolutionary & environmental sciences

For a reference copy of the document with all sections, see nature.com/documents/nr-reporting-summary-flat.pdf

Life sciences study design

All studies must disclose on these points even when the disclosure is negative.

Sample size	We collected three data sets. (1) We collected a comprehensive set of 219 non-redundant multi-domain proteins with sequence identities < 30% to test the developed pipeline. (2) We collected 164 multi-domain proteins from AlphaFold database (https://alphafold.ebi.ac.uk/) to evaluate the performance of DeepAssembly. (3) We collected 247 heterodimeric protein complexes with known interfaces to test the performance of DeepAssembly on protein complex prediction. These complexes share <30% sequence identity and have a resolution between 1-5 Å.
Data exclusions	No data were excluded from the analyses.
Replication	All attempts at replication were successful.
Randomization	n/a
Blinding	There is no blinding group in the experiment, as all proteins in our test have the deposited models in the PDB.

Reporting for specific materials, systems and methods

We require information from authors about some types of materials, experimental systems and methods used in many studies. Here, indicate whether each material, system or method listed is relevant to your study. If you are not sure if a list item applies to your research, read the appropriate section before selecting a response.

Materials & experimental systems

n/a	Involved in the study
☒	☐ Antibodies
☒	☐ Eukaryotic cell lines
☒	☐ Palaeontology and archaeology
☒	☐ Animals and other organisms
☒	☐ Clinical data
☒	☐ Dual use research of concern
☒	☐ Plants

Methods

n/a	Involved in the study
☒	☐ ChIP-seq
☒	☐ Flow cytometry
☒	☐ MRI-based neuroimaging
